# *Babesia* spp. in Domestic Animals from Rural Areas of Cauca Department: Previous Exposure and Molecular Detection Among Canines, Bovines and Equines

**DOI:** 10.1007/s11686-025-01156-2

**Published:** 2025-11-14

**Authors:** Carlos Ramiro Silva-Ramos, Juan Andrés Niño Rodríguez, Juliana Gil-Mora, Paola Betancourt-Ruiz, Heidy- C. Martínez-Díaz, Elkin Forero-Becerra, J. Manuel Matiz-González, Eliana Bolaños, Luz-Adriana Olaya-M, Efraín Benavides, Marylin Hidalgo

**Affiliations:** 1 Grupo de Enfermedades Infecciosas, Departamento de Microbiología, Facultad de Ciencias, Pontificia Universidad Javeriana, Carrera 7 No. 43-82, Bogotá, Bogotá D.C. Colombia; 2 Centro de Investigaciones Odontologicas, Facultad de Odontología, Pontificia Universidad Javeriana, Bogotá, Colombia; 3 Universidad de Ciencias Aplicadas y Ambientales, Bogotá, Colombia; 4 Molecular Genetics and Antimicrobial Resistance Unit, Universidad El Bosque, Bogotá, Colombia; 5 Secretaría Departamental de Salud, Atlántico, Colombia; 6 Universidad Libre, Seccional Cali, Cali, Colombia; 7 Grupo Epidemiología y Salud Pública, Facultad de Ciencias Agropecuarias, Universidad de La Salle, Bogotá, Colombia

**Keywords:** Babesia, Canines, Bovines, Equines, Serology, Molecular detection, Phylogeny, Colombia

## Abstract

**Purpose:**

*Babesia* species are tick-borne protozoan parasites which affect several animal species. Babesia spp. infections are significantly important for veterinary medicine, affecting a wide range of domestic animal species such as dogs, cattle, and horses. In Colombia, studies of *Babesia* spp. infections in domestic animals are scarce. Thus, the aim of the present study was to explore the circulation of these parasites among domestic canines, bovines and equines from the department of Cauca.

**Methods:**

Between August and November, 2017, active domestic animal sampling of cattle was performed in eight rural areas of four municipalities of Cauca department. Serum and whole-blood samples were obtained from all specimens for serological and molecular tests. Immunofluorescence assays were performed on all serum samples to detect antibodies against *Babesia* spp., and DNA was extracted from whole-blood samples to perform a genus-specific PCR to identify the presence of *Babesia* spp.

**Results:**

A total of 198 samples were collected: 52.5% from dogs, 32.3% from horses, and 15.2% from cattle. Seroprevalence rates showed that 58.1% of domestic animals were exposed to *Babesia* spp., with the highest rates among equines (65.6%). Molecular detection revealed that 17.7% had an active *Babesia* spp. infection, being more frequent among cattle (53.3%). Phylogenetic analysis indicated that *Babesia bigemina* was the primary species identified.

**Conclusion:**

This study provides critical data on *Babesia* infections in domestic animals in Cauca department, revealing active infections and previous exposures among domestic animals from the region.

## Introduction

*Babesia* spp. is a parasitic genus which includes hemoprotozoan microorganisms transmitted by ticks that can infect a wide range of mammalian and avian hosts [1]. Currently, more than 100 *Babesia* species have been officially described, but only six of them and two genetic variants are known to be pathogenic for humans. They are transmitted by anthropophilic ixodid ticks of the *Ixodes ricinus* complex, *Dermacentor albipictus* and *Ixodes ovatus* [2, 3]. Human babesiosis has been officially described only in the northern hemisphere, with more cases reported from the northeastern part of the United States [3]. The non-specific febrile illness similar to malaria can range from a mild or even asymptomatic infection, to a severe and fatal disease due to multiorgan failure, mainly in patients with immunocompromise or comorbidities [2, 4].

Although *Babesia* spp. is important for human health, this parasitic genus has greater relevance for veterinary medicine since several *Babesia* species are well-known pathogens of a wide range of domestic animal species including dogs, cattle and horses [5–7].

Canine babesiosis has a widespread distribution in tropical regions all across the world and is known to be caused by six different *Babesia* spp.: *B. canis*, *B. vogeli*, *B. rossi*, *Babesia gibsoni*, *B. conradae* and *B. vulpes*. *Babesia canis* and *B. gibsoni* are the traditionally recognized causative agents, and *Babesia vogeli* the most widespread species. They are transmitted by ticks of the *Dermacentor*, *Haemaphysalis* and *Rhipicephalus* genera [8–10]. In Latin America and the Caribbean, canine babesiosis molecular prevalence is variable and depends on the region and studied population, ranging from 0.9 to 21.7% [10]; however, previous exposure and seropositivity can be as high as 57.9% [11].

In equine species such as horses, donkeys, zebras and mules, *Babesia* infection is known as equine piroplasmosis or equine babesiosis, which are parasitic tick-borne diseases caused by *B. caballi* and *Theileria equi* (formerly *B. equi*). These *Babesia* spp. are transmitted by tick species belonging to several genera including *Hyalomma*, *Rhipicephalus* and *Dermacentor* [6]. Equine piroplasmosis can be severe and potentially life-threatening for individuals that are not promptly diagnosed and treated [12]. It is a major concern for the equid industry since its impact on domestic equines’ health decreases the performance and productivity of domestic equines [13]. In Latin America, equine piroplasmosis is highly prevalent with seroprevalence rates around 58% and molecular prevalence ranging between 15.9% and 56.9% [14].

Bovine babesiosis is an important parasitic tick-borne infectious disease that affects cattle and other bovine species which is caused by six different *Babesia* species: *B. bigemina*, *B. bovis*, *B. divergens*,* B. major*,* B. occultans* and *B. argentina*. *Babesia bigemina* and *B. bovis*, the most frequent ones, are transmitted mainly by *Rhipicephalus* (*Boophilus*) *microplus* and *R. annulatus* ticks [5, 15]. The disease can be mild, but severe cases are not uncommon, leading to substantial economic losses for the livestock industry, being particularly challenging to manage due to the impact generated on the cattle herd’s health and productivity [16]. Globally, bovine babesiosis is an important veterinary problem, and South America has been postulated as the region with the highest prevalence for this tick-borne disease [17].

In Colombia few studies conducted on *Babesia spp.* and domestic animal species such as canines, bovines and equines have shown different serological and molecular prevalence rates, depending on the region [18–20]. However, most of these studies have been conducted in economically important regions where these diseases represent an important problem for animal productivity [18, 19, 21]. This leaves aside other regions where canine, bovine and equine health is not a priority, such as the department of Cauca, where no studies have been conducted to date. Thus, the aim of the present study was to explore the circulation of these parasites among domestic canines, bovines and equines from four rural areas of the department of Cauca through serological and molecular approaches to shed light on the infection rates among these animal populations.

## Materials and methods

### Study Area

Cauca department (00°58’54” and 03°19’04” N, 75°47’36” and 77°57 × 05” W) is one of the 32 departments of Colombia. It is located in the southwest of the country, within the Andean and Pacific regions; and has a total area of 29.308 km^2^ which represents 2.56% of the national territory. Cauca department is divided into 42 municipalities which are grouped into five subregions: north (Buenos Aires, Caloto, Corinto, Guachené, Miranda, Padilla, Puerto Tejada, Santander de Quilichao, Suárez and Villa Rica), south (Almaguer, Argelia, Balboa, Bolívar, Florencia, La Vega, Mercaderes, Patía, Piamonte, San Sebastián, Santa Rosa and Sucre), east (Caldono, Inzá, Jambaló, Páez, Puracé, Silvia, Toribío and Totoró), west (Guapí, López de Micay and Timbiquí), and center (Cajibío, El Tambo, La Sierra, Morales, Piendamó, Popayán, Rosas, Sotará and Timbio). The average temperature in Cauca department varies between 8 and 28 °C, which is determined mainly by its geography with warm tropical humid regions on the Pacific coast and the center of the department and cold climate in the eastern region. The vegetation cover includes dense upper layers of trees and shrubs that provide organic matter through leaf litter, and lower grass layers that generate a humid microclimate ideal for biological diversity. It has a mean altitude of 1693 m above sea level with an annual mean precipitation exceeding 7000 mm in most of the department with minimal or absent dry seasons (https://www.cauca.gov.co accessed on April 7th, 2025). Agriculture is the main economic activity in the region; however, livestock and fish farming have also been gaining notable development in recent times. According to the 2018 national population and housing census of the “Departamento Administrativo Nacional de Estadística - (DANE)”, Cauca department has a total population of 1,243,503 inhabitants, of which 492,229 live in the main urban areas, 131,451 in suburban settlements and 619,823 in rural areas (https://www.dane.gov.co accessed on April 7th, 2025); it has the largest indigenous population in Colombia. To date, six tick species have been identified to be actively circulating in Cauca department, including *Amblyomma patinoi*, *Dermacentor nitens*, *Ixodes auritulus*, *I. montoyanus*, *R. microplus*, and *R. sanguineus* s.l [22, 23].

### Samples

Between August and November 2017, active domestic animal random sampling including apparently healthy dogs (*Canis lupus familiaris*), horses (*Equus caballus*), and cattle (*Bos taurus* and *Bos indicus*) was performed in eight rural areas of four municipalities of Cauca department (Caloto, El Tambo, La Sierra and Santander de Quilichao). Procedures involving animals and permits for sampling were approved on May 11th, 2016, by the Ethics and Research Committee of the Faculty of Sciences of the “Pontificia Universidad Javeriana”. Signed consent for sampling was voluntarily obtained from each domestic animal’s owner. Dogs, horses and cattle were manually restrained with the help of the owners, and then a whole-blood and serum sample were collected via cephalic venipuncture in ethylenediaminetetraacetic acid (EDTA) and dry tubes, respectively, and stored at −80 °C at Pontificia Universidad Javeriana’s special bacteriology laboratory until further processing.

### Detection of Previous Exposure to *Babesia* spp.

Detection of antibodies against *Babesia* spp. in domestic animals was evaluated through indirect immunofluorescence assay (IFA) using three commercial kits from Fuller Laboratories (Fullerton, CA, USA): *B. canis* IFA Kit for dogs, *B. caballi* IFA Kit for horses, and *B. bovis* IFA Kit for cattle. All procedures were performed using the manufacturer’s instructions. Serum samples were initially evaluated in 1:50, 1:80 and 1:40 dilutions in phosphate-buffered saline (PBS) solution for dogs, horses and cattle, respectively. A positive (reactive dog, horse and bovine serum) and a negative control (non-reactive dog, horse and bovine serum) were provided in each commercial kit and evaluated at the same dilution mentioned above.

### DNA Extraction

DNA was extracted from 200 µL of each animal; whole-blood sample using the DNeasy^®^ Blood & Tissue Kit (250) (Qiagen, Hilden, Germany) according to the manufacturer’s instructions. After each extraction procedure, to assess the DNA sample integrity and rule out the presence of inhibitors, DNA was quantified spectrophotometrically (West Tune NanoGenius series, Hangzhou, China) and its quality was evaluated through end-point PCR targeting a 289-base pair fragment of the β-actin encoding gene (*ACTB*) using primers XAHR 17 (CGGAACCGCTCATTGCC) and XAHR 20 (ACCCACACTGTGCCCATCTA) [24] under the following conditions: one cycle for 4 min at 95 °C; 35 cycles for 30 s at 94 °C, 30 s at 57 °C, and 60 s at 72 °C; and a final extension for 5 min at 72 °C. Infected canine whole-blood DNA positive sample for *Babesia* spp. obtained in a previous research project and sterile molecular grade water were included as positive and negative controls, respectively, for each amplification procedure. Subsequently, amplicons were visualized in an 1% agarose gel stained with SYBR™ Safe DNA gel Stain (Invitrogen, Waltham, MA, USA) and separated by electrophoresis.

### Detection of *Babesia* spp. DNA

To determine the presence of *Babesia* spp. DNA among domestic animals, whole-blood samples, a genus-specific end-point PCR was performed targeting an approximately 650-bp fragment of the *18 S rRNA* gene of *Babesia* spp. using the primers Bab-F (GTGAAACTGCGAATGGCTCA) and Bab-R (CCATGCTGAAGTATTCAAGAC) [25] under the following conditions: one cycle for 3 min and 30 s at 95 °C; 34 cycles for 30 s at 95 °C, 30 s at 59 °C, and 90 s at 72 °C; and a final extension for 5 min at 72 °C, together with PowerUp™ SYBR™ Green Master Mix (Applied Biosystems, Austin, TX, USA). A negative control, similar to *ACTB* protocol, and a positive control, *B. canis* DNA obtained in a previous study from a canine infected with *Babesia* spp., were used for all PCR reactions. Amplicons were visualized in 1% agarose gel stained with SYBR™ Safe DNA gel Stain (Invitrogen, Waltham, MA, USA), and then were purified and bi-directionally sequenced for phylogenetic analysis.

### Phylogenetic Analyses

Successfully sequenced samples and *Babesia* spp. reference sequences, retrieved from NCBI GeneBank database, were aligned using the ClustalW algorithm [26], and then, analyzed by phylogenetic inference using the Maximum-Likelihood method [27]. Evolutionary distances for *Babesia* spp. *18 S rRNA* gene alignments were calculated using the GTR model [28, 29], which is the best model according to the Bayesian information criterion, with 1000 bootstrap replicates. All positions containing gaps and missing data were excluded, and the analysis was performed in MEGA software, version 10 [30].

### Statistical Analysis

Serological and molecular prevalence rates of *Babesia* spp. among domestic animals from Cauca department were calculated along with their corresponding 95% confidence intervals (CI). Differences in prevalence among domestic animal species and municipalities were evaluated using the Pearson’s chi-square test, or the Fisher’s exact test when the expected frequencies were lower than five. All statistical analyses were performed using JASP 0.16.3 statistical software (JASP Team, Amsterdam, Netherlands) for Windows. A *p*-value result < 0.05 was considered statistically significant.

## Results

### Samples

A total of 198 domestic animal samples were collected between August and November of 2017 in eight villages from four municipalities of Cauca department. Of these, 52.5% (*n* = 104) of the samples were collected from dogs, 32.3% (*n* = 64) were from horses and 15.2% (*n* = 30) were from cattle. Regarding geographic origin, 47.5% (*n* = 94) samples were collected in El Tambo municipality, 30.3% (*n* = 60) in Santander de Quilichao, 15.6% (*n* = 31) in Caloto, and the last 6.6% (*n* = 13) samples were collected in La Sierra municipality.

### Detection of Previous Exposure to *Babesia* spp.

**Fig. 1:**
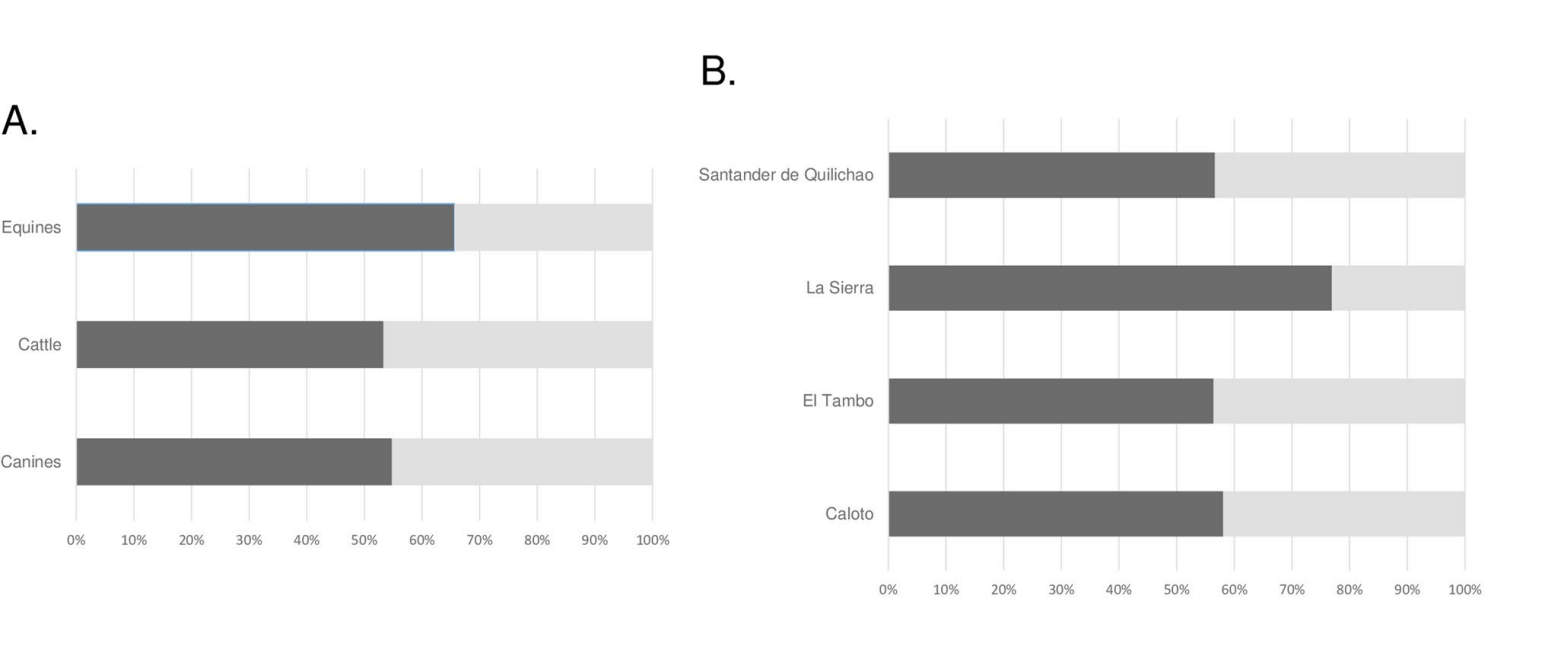
Seroprevalence rates according to **A** animal species and **B** geographic origin. Each bar represents one species, and the segments show the proportion of seropositive (black bar) and seronegative (light-gray bar) individuals within each group

Table 1 highlights the serologic results according to the geographical origin of the animals. Previous exposure to *Babesia* spp. was identified in 58.1% (*n* = 115; IC 95%: 51.2–64.8%) domestic animals sampled, of which, the highest seropositivity was detected in equines (65.6% [*n* = 42; IC 95%: 53.3–76.5%]), followed by canines (54.8% [*n* = 57; IC 95%: 44.9–64.2%]) and cattle (53.3% [*n* = 16; IC 95%: 34.3–1.7%]) (Fig. 1A). According to sample geographic origin, 76.9% (*n* = 10; IC 95%: 46.2–94.9%) seropositive samples were identified in La Sierra municipality, 58.1% (*n* = 18; IC 95%: 39.1–75.0%) in Caloto, 56.7% (*n* = 34; IC 95%: 43.1–69.4%) in Santander de Quilichao, and 56.4% (*n* = 53; IC 95%: 45.8–66.5%) in El Tambo municipality. Equines from La Sierra (87.5% [*n* = 7]) and Santander de Quilichao (84.6 [*n* = 11]) municipalities, and canines from Caloto (66.7% [*n* = 18]) municipality, showed the highest seropositive rates (Fig. 1B). No statistically significant differences were observed in seroprevalence rates between animal species (*p* = 0.3276) or among municipalities (*p* = 0.4932).

**Table 1:** Seropositivity results against *Babesia* spp. Among dogs, cattle and horses from four municipalities of Cauca department

Municipality	Village	Canines			Bovines			Equines		
		Samples	Seropositive	Prevalence (%)	Samples	Seropositive	Prevalence (%)	Samples	Seropositive	Prevalence (%)
Caloto	El Credo	19	11	57.9	None	None	None	1	0	0
	Huellas	8	7	87.5	None	None	None	3	0	0
	Total	27	18	66.7	None	None	None	4	0	0
El Tambo	Betania	9	4	44.4	4	2	50	10	7	70
	Placer	17	9	52.9	1	1	100	11	6	54.5
	Zarzal	14	7	50	10	6	60	18	11	61.1
	Total	40	20	50	15	9	60	39	24	61.5
La Sierra	Juana Castaña	5	3	60	None	None	None	8	7	87.5
	Total	5	3	60	None	None	None	8	7	87.5
Santander de Quilichao	Lomitas abajo	15	11	73.3	12	6	50	5	3	60
	Lomitas arriba	17	5	29.4	3	1	33.3	8	8	100
	Total	32	16	50	15	7	46.7	13	11	84.6
Total		104	57	54.8	30	16	53.3	64	42	65.6

### Detection of *Babesia* spp. DNA

**Fig. 2:**
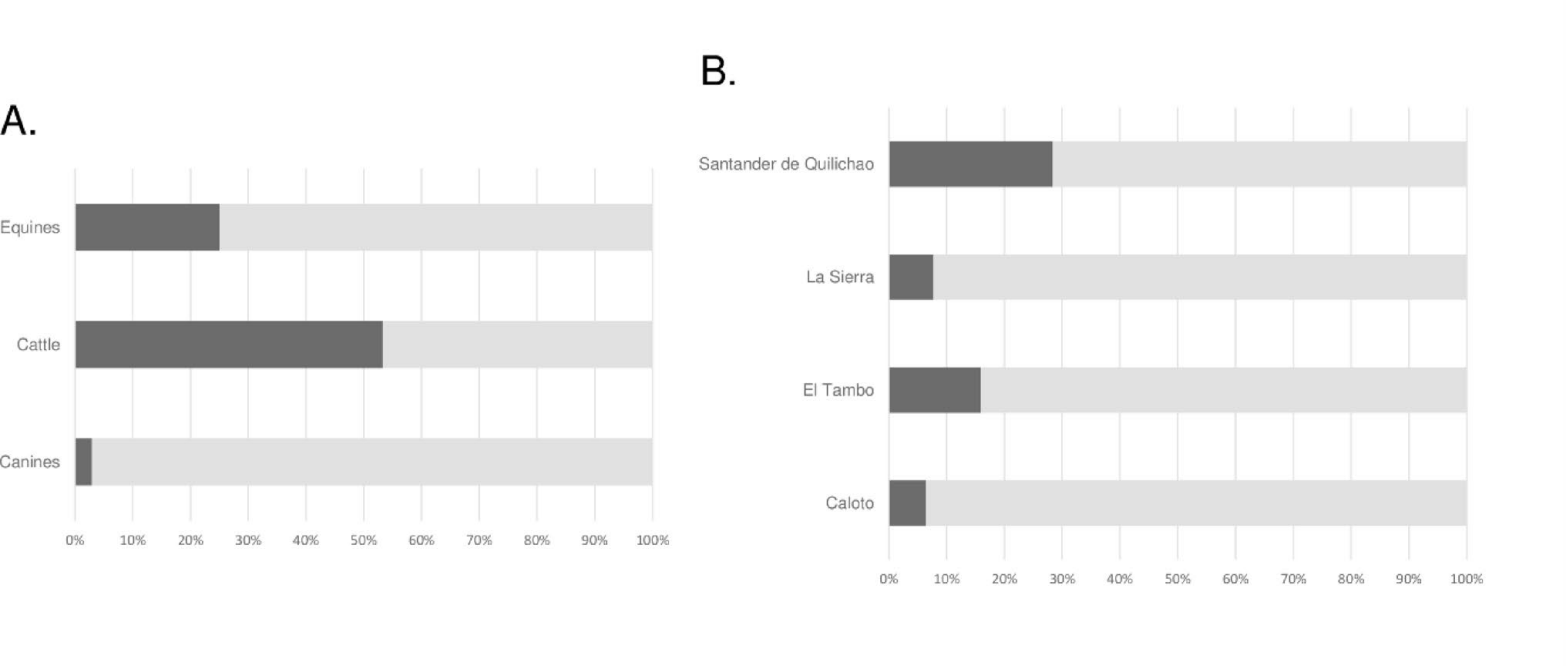
Molecular prevalence rates according to **A** animal species and **B** geographic origin. Each bar represents one species, and the segments show the proportion of seropositive (black bar) and seronegative (light-gray bar) individuals within each group

The molecular results according to the geographical origin of the animals are presented in Table 2. The presence of *Babesia* spp. was detected in 17.7% (*n* = 35; IC 95%: 12.8–23.9%) of domestic animals sampled. Specifically, *Babesia* spp. was detected in 53.3% (*n* = 16; IC 95%: 34.3–71.7%) of sampled cattle, 25% (*n* = 16; IC 95%: 15.7–36.6%) of horses, and only 2.9% (*n* = 3; IC 95%: 0.6–8.3%) of dogs (Fig. 2A). According to geographic origin, 28.3% (*n* = 17; IC 95%: 17.6–41.1%) of positive samples were identified in Santander de Quilichao municipality, 16% (*n* = 15; IC 95%: 9.4–25.1%) in El Tambo, 7.7% (*n* = 1; IC 95%: 0.4% – 33.3%) in La Sierra, and 6.5% (*n* = 2; IC 95%: 1.1–21.4%) in Caloto municipality. The highest frequencies of infection were detected among horses (61.5% [*n* = 8]) and cattle (60% [*n* = 9]) from Santander de Quilichao municipality, and cattle (46.7% [*n* = 7]) from El Tambo municipality (Fig. 2B). Statistically significant differences were found in molecular prevalence rates between species (*p* = < 0.0001) and among municipalities (*p* = 0.0376).

**Table 2:** Molecular detection of *Babesia* spp. DNA among dogs, cattle and horses from four municipalities of the Cauca department

Municipality	Village	Canines			Bovines			Equines		
		Samples	Positive	Prevalence (%)	Samples	Positive	Prevalence (%)	Samples	Positive	Prevalence (%)
Caloto	El Credo	19	2	10.5	None	None	None	1	0	0
	Huellas	8	0	0	None	None	None	3	0	0
	Total	27	2	7.4	None	None	None	4	0	0
El Tambo	Betania	9	0	0	4	4	100	10	2	20
	Placer	17	0	0	1	0	0	11	6	54.5
	Zarzal	14	0	0	10	3	30	18	0	0
	Total	40	0	0	15	7	46.7	39	8	20.5
La Sierra	Juana Castaña	5	1	20	None	None	None	8	0	0
	Total	5	1	20	None	None	None	8	0	0
Santander de Quilichao	Lomitas abajo	15	0	0	12	8	66.7	5	2	40
	Lomitas arriba	17	0	0	3	1	33.3	8	6	75
	Total	32	0	0	15	9	60	13	8	61.5
Total		104	3	2.9	30	16	53.3	64	16	25

### Phylogenetic Analyses

**Fig. 3:**
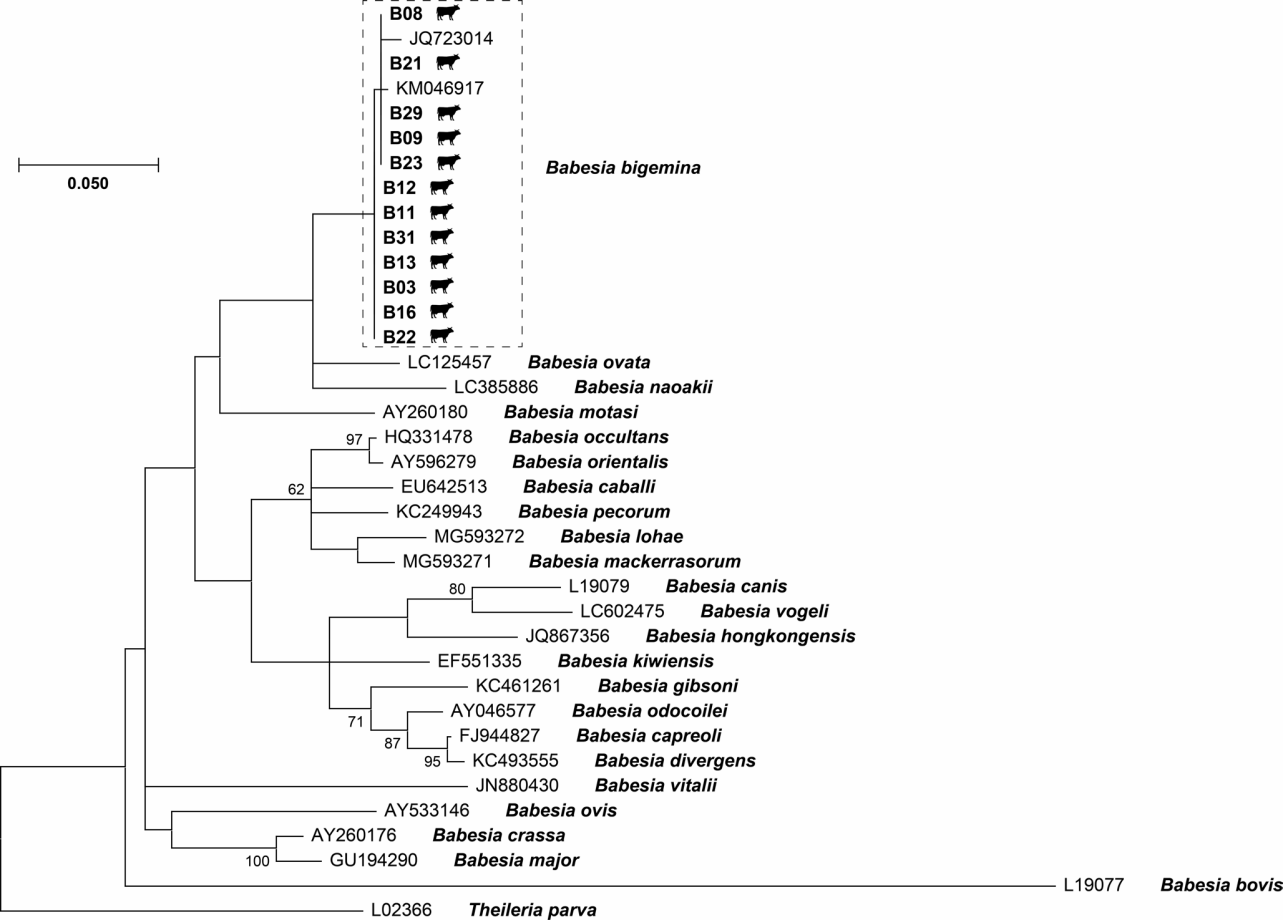
Maximum-likelihood phylogeny of *Babesia* spp. *18 S rRNA* from bovines and representative *Babesia* species. Bovine-related *Babesia* are signaled by the bovine icons. GenBank accession of the reference *Babesia 18 S rRNA* sequences are shown, and the *Babesia bigemina* clade is indicated by a dashed box. Only bootstraps values higher than 50% are shown

To identify the possible *Babesia* species detected among domestic animals from rural areas of Cauca department, a maximum-likelihood phylogeny using the *Babesia* spp. *18 S rRNA* gene was developed. All sixteen and three amplicons obtained from cattle and dogs, respectively, were sequenced, but only twelve good-quality sequences detected from cattle were retrieved and used for further phylogenetic analysis. All twelve cattle-related *Babesia* spp. sequences clustered together forming a clade with reference sequences of *B. bigemina* (Fig. 3), showing a 99.5–100% identity and 100% coverage with one of the reference sequences (KM046917), which has been identified as *B. bigemina* isolate Swiss_6 [31]. None of the sixteen amplicons detected from horses were sequenced due to economical limitations.

## Discussion

Cauca department has become an interesting region for the circulation of several tick-borne pathogens such as *Anaplasma*, *Ehrlichia*, *Rickettsia* and *Borrelia* spp. among different domestic animal species [22, 32]. This situation highlights the importance of tick-borne pathogens for animal health in the region. However, in none of these studies has circulation of *Babesia* species been assessed despite its great importance for animal health. The climatic and geographic diversity of Cauca, characterized by high rainfall, warm lowland areas, and cooler highland regions, may influence the ecology and distribution of tick vectors and *Babesia* spp. Warmer and more humid environments favor tick survival and pathogen transmission, potentially impacting infection rates among domestic animals [33]. In this way, the present manuscript gives the first data on the active circulation of *Babesia* spp. among domestic animal species in four municipalities of Cauca department, detecting exposure and infections with *Babesia* spp. in dogs, cattle and equines from the region. Serological and molecular prevalence rates detected may have been influenced by the circulation of a competent tick vector, animal management practices (e.g., free-ranging animals) and close interspecies interactions within rural communities. These factors may have facilitated vector-host contact, and thus, pathogen transmission, although specific data assessment were beyond the scope of the present study.

In Latin America and the Caribbean *B. vogeli* and *B. gibsoni*, have been detected to be actively circulating in several regions [10]. In addition on unconventional *Babesia* species such as *B. caballi* has been detected in a dog in Brazil [34]. In Colombia, few studies have been performed on canines, identifying seroprevalence rates as high as 89.7% and 83.9% among dogs from Bucaramanga (Santander department) and Villavicencio (Meta department) municipalities, respectively [20], showing that dogs are highly exposed to *Babesia* species in these regions, possibly others such as Cauca department in which through the present study, a seroprevalence of 54.8% was observed. A few molecular studies have also identified a molecular prevalence ranging between 4.5% among dogs from Santamarta municipality (Magdalena department) [35] to 15% among dogs from Pereira municipality (Risaralda department) [36]. In the present study, 2.9% of sampled dogs in Cauca department were found to be infected with *Babesia* spp., but unfortunately, the specific *Babesia* species could not be identified. However, given that previous studies have identified *B. vogeli* as the species involved in other regions of Colombia [20, 35–37], it is likely that this *Babesia* sp. might be the cause of *Babesia* infection in canines in Cauca department. Further studies are needed to confirm this hypothesis.

South America is one of the main regions worldwide where a high seroprevalence (around 58%) and molecular prevalence (ranging between 15.9 and 56.9%) of both pathogens has been detected among domestic equines [14]. Brazil is the main country where equine piroplasmosis represents a recognized important veterinary problem [38–40]. In Colombia few studies have been conducted on *Babesia spp.* in equines. The first one performed in 1988 detected high seroprevalences of 90 and 94% against *B. caballi* and *T. equi* among equines in Cordoba department [41]; and more recently, studies revealed a prevalence of 18.3% by microscopy among horses from the same region [42]. A molecular prevalence of 13.5% among equines from several regions from Antioquia department [43], and of 25.7% among horses from Arauca, Meta and Santander departments were documented with the identification of *T. equi* [44]. In our study, a high seroprevalence of 65.6% and a molecular prevalence of 25%, determined that equines from these regions are highly infected by *Babesia* spp. Unfortunately, due to financial limitations, we were unable to perform sequencing analysis of these equine positive samples; thus, future studies should aim to provide data regarding the circulating piroplasm species infecting horses in this region to assess the prevalence of infection among equines and identify the implicated tick vector species.

It is important to note that the low molecular detection rates observed in dogs and horses may be attributed to several biological, but also methodological, factors such as low parasitemia levels, transient presence of *Babesia* spp. in the peripheral blood, or limitations in the sensitivity of the PCR method used for the detection. It is possible that these factors could have led to underestimation of the true infection rates in these animals. Additionally, since Cauca department is endemic for other tick-borne pathogens such as *Anaplasma* and *Ehrlichia* [22, 32], co-infections can occur and may be more common than expected. Although this study did not investigate coinfections, previous reports of high tick infestation rates in Cauca department [22] suggest that concurrent infections are probable and should be addressed in future research.

Finally, bovine babesiosis is an important threat not only for veterinary medicine but also for the local economy since this tick-borne disease affects the health and the production of cattle reducing the milk and meat production, and increasing morbidity and mortality [15]. Global prevalence of bovine babesiosis has been estimated at 29%, with an active infection of 16% and a previous exposure of 50% [17]. South America is the region with the highest prevalence for bovine babesiosis worldwide [17, 45], with results as high as 80% in Guyana [46], 79% in Paraguay [47] and 66% in Bolivia [48], emphasizing that bovine babesiosis is an important problem for livestock in the region. Regarding Colombia, several studies have been conducted, most of them focused on Antioquia department and the Orinoquía region which are the main Colombian livestock regions [18, 19, 21, 49, 50]. These studies have demonstrated high molecular prevalences such as 83.9% among cattle from the Magdalena medio region in Antioquia department [18], as well as seroprevalence of 55.4% among specimens from the Urabá antioqueño region [49]. To our knowledge, this is the first time that *Babesia* infection in cattle has been investigated in the Cauca department, revealing a serological and molecular prevalence of 53.3% among the sampled cattle. Sequences derived from bovine samples identified *B. bigemina* as the predominant *Babesia* species present in the region, consistent with its widespread circulation across South America [17, 45]. Studies performed in Colombia have also determine that this *Babesia* sp. and *B. bovis* are the circulating species in several regions [19, 21]. Additional studies have also identified the presence of one of these *Babesia* spp., *B. bigemina*, in *Rhipicephalus* (*Boophilus*) *microplus* specimens attached to cattle from a region in the department of Antioquia [51]. This tick species is a recognized vector of *B. bigemina*, but it can also transmit several other pathogens, such as *Anaplasma marginale*, the agent of bovine anaplasmosis which has also been identified infecting cattle in Colombia [49, 52]. This complicates the implementation of effective prevention and control measures in the country, hindering efforts to improve livestock production. By implementing molecular markers complementary to *18 S rRNA* gene, such as *rap-1c*, it is possible to characterize the intraspecific groups circulating in the region, as this marker has a greater capacity to resolve genetic groups within the species [53].

It is important to note that the seroprevalence rates observed in our study were higher than the molecular detection rates. This is consistent with the natural history of *Babesia* spp. infections, where animals can remain seropositive for prolonged periods after clearing the active infection. In contrast, PCR only detects active infections at the time of sampling. Therefore, the higher seropositivity reflects previous exposures to *Babesia* spp., while the lower molecular prevalence may indicate that only a fraction of animals were actively infected during the study period.

However, the discrepancy observed between serological and molecular results deserves further consideration. This can also be explained by the host immune response, since cattle, dogs and equines can also develop chronic or subclinical infections and remain seropositive long after parasitemia decreases below the PCR detection threshold. Additionally, all tick vector stages can transmit *Babesia* spp., leading to repeated exposures and therefore long-lasting antibody responses.

In addition, it is important to consider that serological methods such as IFA may present cross-reactions between different *Babesia species*. Antibodies generated against one species can recognize antigens of closely related species. This phenomenon has been well documented in areas where multiple *Babesia* species co-circulate, leading to high seropositivity despite the detection of low molecular prevalence. Such cross-reactivity may partly explain the higher serological prevalence compared to PCR detection in our study, where animals may have been exposed to more than one *Babesia* species or retained long-lasting antibodies from previous infections.

Given the detection of *Babesia* spp. infections among domestic animals, it is important to implement integrated tick control programs, including the regular use of acaricides, pasture management practices to reduce tick habitats, and routine veterinary check-ups. Additionally, awareness campaigns targeting animal owners are crucial to enhance early detection and prevention of tick-borne diseases, ultimately protecting both animal health and local economies.

This study has several limitations. First, sequencing could not be performed on positive samples from equines and canines due to financial limitations, limiting species-level identification in these hosts. Second, the sampling strategy was based on convenience sampling and did not stratify by farm types or management practices, preventing an evaluation of risk factors associated with *Babesia* spp. infection. Third, environmental variables such as tick abundance, vegetation types, and climate parameters were not systematically recorded, which could influence vector dynamics and pathogen distribution. Fourth, co-infections with other tick-borne pathogens were not assessed, despite their epidemiological relevance. Finally, the modest sample size, particularly when stratified by species and municipality, may have limited the statistical power to detect significant associations. Future research should incorporate larger, stratified sampling designs, environmental data collection, and molecular detection of multiple pathogens to achieve a more comprehensive epidemiological analysis.

Considering the tropical climatic conditions and the ecological similarities with other Colombian regions, it is plausible that the department of Cauca presents a situation of endemic stability for *Babesia* spp. In such settings, continuous tick exposure from early life induces the development of long-lasting partial immunity in the host population, which may explain the reduction in the incidence of the disease and thus, clinical cases are uncommon despite ongoing transmission. This epidemiological scenario is compatible with our findings of high seroprevalence but comparatively lower molecular detection, since animals can remain immune and seropositive without harboring detectable parasitemia. Moreover, in areas where multiple *Babesia* species co-circulate, repeated exposures generate cross-reactive antibody responses that further increase seroprevalence values. Therefore, the high serological prevalence observed in Cauca does not contradict the concept of endemic stability, but rather reflects the combined effect of acquired immunity and immunological cross-reactions resulting from multiple exposures to different *Babesia* species. Future studies should evaluate the epidemiological thresholds and clinical impact of *Babesia* spp. infections in this region to confirm this hypothesis.

## Conclusion

The present study documents, for the first time, the active circulation and previous exposure to *Babesia* spp. among domestic animals (canines, bovines and equines) in Cauca department, Colombia, highlighting a scenario of high seroprevalence and notable molecular prevalence, particularly in cattle. The molecular confirmation of *B. bigemina* as the circulating species in bovines emphasizes the need for greater attention to tick-borne diseases in the region. These findings reinforce the significance of babesiosis for veterinary medicine because of its potential impact on local animal health and livestock production. Future research should aim to characterize the specific *Babesia* species infecting dogs and horses, identify tick vectors involved in transmission of these *Babesia* species, and evaluate the clinical and economic consequences of these infections. Strengthening surveillance efforts could also have important implications for public health, considering the close interactions between domestic animals and human communities in rural areas.

## Data Availability

No datasets were generated or analysed during the current study.
